# The Sulphites in Zymotic Diseases

**Published:** 1868-05

**Authors:** P. G. Kelsey


ARTICLE XV.

### THE SULPHITES IN ZYMOTIC DISEASES.

BY P. G. KELSEY, M.D.

An Extract from an Essay read before the Allen Co. Medical Association, held at Fort Wayne, Ind., April 7th, 1868.

* * * * The use of the sulphites and hyposulphites in the treatment of zymotic diseases has attracted considerable attention of late, on the part of the profession, and very worthily I think, and having tested their virtues to some extent in my own practice with happy results, I will, with your permission, cite a few cases by way of testimony. I shall, at present, refer more especially to the use of the *sulphite of soda* in variola, and not to detain you by indulging in a *preamble*, referring to authority, etc., I will give you the cases at once.

CASE I. E. S., soldier, home on furlough, *æt* 23, was taken sick March 11th, 1864, with fever, severe headache, cough, and pain in chest, which he supposed was the result of exposure before coming home. Was not aware of any exposure to *small-pox* and, of course, did not expect it. I was called the following day to see him, and the third day thereafter a bountiful eruption, of an unmistakable character, decided all questions of doubt, the entire body being covered as completely with the eruption of *variola* as it well could be, and the disease maintain the distinct form. During the first three days of the eruption, until it reached the vesicular stage, the usual course of treatment was followed. But on the *fourth* day of the eruption, at the suggestion of my then colleague but former excellent preceptor, Dr. Terry, who had noticed some account of its use, I commenced the use of the sulphite of soda, using the following formula:

℞. Soda sulphis,---------------------------- ʒiv.

Syr. Glycyrr.,------------------------ ℥iij.

Aqua,---------------------------------- ℥j.

M.

Of this mixture I ordered a teaspoonful every four hours, and with the result of rendering my patient far more comfortable in every particular, and apparently modifying the disease quite a considerable in the following 24 hours. Notwithstanding the advanced stage of the disease at the time of commencing this treatment, I am certain that through some agency the attack, which at the outset had presented the appearance of a severe case, though retaining the distinct form, was rendered comparatively mild and the pustular stage considerably shortened, some of the later vesicles aborting without going on to suppuration, so that my patient escaped with only a moderate pitting.

CASE II. Though not next in order in my diary, yet, from its relation to the foregoing case, I will next give that of an infant, female, which was in the house and exposed to the above case. The child was but six weeks old at the time of exposure, from which fact, together with that that the disease is not generally considered as easily communicable during the primary stages, I refrained from vaccinating the babe, although this was promptly attended to with all other inmates of the household, who were, also, immediately sent away, save one lady, a sister-in-law, who remained to attend to the young man. Since all escaped without contracting the disease, you can imagine my surprise when the babe was brought back completely covered with small-pox eruption from the crown of its head to the soles of its feet, both inclusive, so that there was not a spot on the child's body where you could rest the end of your finger without touching a pock. For two days the child's mouth was so bad that it nursed with difficulty, but aside from this, as a general thing, it nursed well and bore its affliction with remarkable ease and quietude, and passed through the severe ordeal of the suppurative stage of small-pox, which so severely taxes the strength and vitality of the adult, making a good and rapid recovery, and with only a few marks of any extent. The only medicine I gave the child was the *sulphite of soda* in the syrup of glycyrr., and a little sweet spirits of nitre for the fever. Now, I believe the cases which terminate favorably in such young infants—from six to eight weeks old—are comparatively rare; and whether this child owes its life to the soda or an unusual amount of vitality is, of course, not for me to say.

Case III. J. N., farmer, German, *æt.* about 30, was taken very sick with the measles, March 13, 1864, and sent for me the following day upon the appearance of the eruption, and upon seeing him I found that what the friends and neighbors had looked upon as measles was, in reality, *confluent small-pox*, and of no moderate pretensions. This man was unfortunate in securing for a nurse a man who did not believe in medicine for small-pox, and who presumed to take the treatment into his own hands, much to the detriment of the patient; for, upon my discovering the fact, and causing a return to my remedy, with increased dose, he again became more comfortable. This man was delirious and entirely blind for *five days*, and could take no nourishment aside from a little gruel or broth, but he made a good recovery, carrying, however, the lasting evidence of his "change of doctors," as he believes. Three of his children, aged *one*, *three*, and *five* years, who proved insusceptible to vaccination, had varioloid or variola, as you please. They took the soda as a prophylactic, and were not confined to the bed at all, paying but little heed to the disease.

During this spring (1864) I treated, in all, twelve or thirteen cases of variola and varioloid, using this remedy, and all with favorable results; gaining quite a reputation as a "small-pox doctor," although I did not pretend to make a *specialty* of it; and my confidence in the sulphite of soda, as a valuable remedy in such cases, increased with each additional test.

Dr. Terry also used these preparations in the treatment of typhoid fever, erysipelas, and other like diseases, during my association with him, and speaks highly of them as remedial agents. One case of hospital gangrene, attended by him, is not without interest:

A soldier was wounded in the battle of Chickamauga, with a minie ball, just below the instep of the left foot, the ball passing transversely across the foot. He was treated in the hospital at Chattanooga until the following March, when he was discharged—up to which time we have no history of the case. When discharged, the surgeon in charge not only despaired of saving the foot, but also his life, and advised and urged immediate amputation. His father, however, determined to bring him home first, and, upon their arrival, immediately sent for Dr. Terry, to amputate the foot. I will give the Doctor's own language: "On my first visit, I found the patient emaciated almost to a skeleton; skin dry and husky; pulse 140 per minute; foot and leg intensely swollen and œdematous, with a large gangrenous ulcer involving the whole dorsum of the foot, with thick, indurated, and shelving edges, the gangrene dissolving the tissues beneath, and emitting a most intolerable odor. The treatment for some time had been flaxseed poultice to the ulcer, with quinine, iron, and whiskey internally. I prevailed upon the parents to defer the operation a few days, while I made an effort to save the limb, and also to satisfy myself whether the boy had recuperative power sufficient to react and survive the operation. I cauterized the gangrenous parts with *potassa fusa*, continued the poultice, and also sprinkled the surface with the *sulphite of lime;* continued the general treatment, adding *sulphite of soda* internally. In three days the offensive odor was entirely gone. The eschar separated kindly, and gangrene reappeared, subsequently, but once, yielding readily to the caustic, leaving a healthy granulating surface." This patient made a good recovery, the wound keeping pace with the improvement of his general health, until, in six weeks, it was entirely healed, and the young man walked on both his feet again, instead of one. This much for conservative surgery.

I could add many other cases, in which I have seen good result from the administration of the sulphites, but time will not permit at present, and I have already occupied your attention quite long enough. A case of typhoid fever now under treatment, has, I think, been greatly benefited by the soda—the delirium passing off, mind becoming clear, fever abating, and tongue becoming moist and soft under its influence. But the results of this case are still in the future. The patient had taken the emulsion of turpentine with beneficial results, but the delirium continued until the administration of the preparation of soda, but this *may* have been incidental.

As to the treatment of variola, if Dr. Tanner's ideas are correct, with regard to all drugs heretofore used, and the sulphite of soda will prove an exception, then it is indeed a blessing at hand. He says: "The less drugs are used in the treatment of small-pox, the better; since they will neither shorten the disease, nor exert any favorable influence upon the eruption." He also pronounces the *Sarracenia purpurea*, or pitcher plant, a failure. From my experience, I feel confident that the *sulphite of soda* not only shortened the disease, but also exerted a *favorable influence on the eruption*, many of the vesicles aborting, or stopping short of the suppurative stage.

But I must refrain from further comment at present, however tempting it be, which, together with the probable effect of these articles, I reserve for consideration at some future time.

